# Abdominal perfusion pressure is critical for survival analysis in patients with intra-abdominal hypertension: mortality prediction using incomplete data

**DOI:** 10.1097/JS9.0000000000002026

**Published:** 2024-08-14

**Authors:** Liang Xu, Weijie Zhao, Jiao He, Siyu Hou, Jialin He, Yan Zhuang, Ying Wang, Hua Yang, Jingjing Xiao, Yuan Qiu

**Affiliations:** a Department of General Surgery, The Second Affiliated Hospital of the Army Medical University; b Bio-Med Informatics Research Centre and Clinical Research Centre, The Second Affiliated Hospital of the Army Medical University; c Bioengineering College, Chongqing University; d Department of Respiratory and Critical Care Medicine, The First Affiliated Hospital of Chongqing Medical University; e Department of Gastroenterology, The Second Affiliated Hospital of the Army Medical University; f Department of General Surgery, Chongqing General Hospital, Chongqing; g Medical Big Data Research Center, Chinese PLA General Hospital, Beijing, People’s Republic of China

**Keywords:** abdominal perfusion pressure, incomplete data, intra-abdominal hypertension, mortality prediction

## Abstract

**Background:**

Abdominal perfusion pressure (APP) is a salient feature in the design of a prognostic model for patients with intra-abdominal hypertension (IAH). However, incomplete data significantly limits the size of the beneficiary patient population in clinical practice. Using advanced artificial intelligence methods, the authors developed a robust mortality prediction model with APP from incomplete data.

**Methods:**

The authors retrospectively evaluated the patients with IAH from the Medical Information Mart for Intensive Care IV (MIMIC-IV) database. Incomplete data were filled in using generative adversarial imputation nets (GAIN). Lastly, demographic, clinical, and laboratory findings were combined to build a 7-day mortality prediction model.

**Results:**

The authors included 1354 patients in this study, of which 63 features were extracted. Data imputation with GAIN achieved the best performance. Patients with an APP <60 mmHg had significantly higher all-cause mortality within 7–90 days. The difference remained significant in long-term survival even after propensity score matching (PSM) eliminated other mortality risks between groups. Lastly, the built machine learning model for 7-day modality prediction achieved the best results with an AUC of 0.80 in patients with confirmed IAH outperforming the other four traditional clinical scoring systems.

**Conclusions:**

APP reduction is an important survival predictor affecting the survival prognosis of patients with IAH. The authors constructed a robust model to predict the 7-day mortality probability of patients with IAH, which is superior to the commonly used clinical scoring systems.

## Graphical abstract

**Figure:**
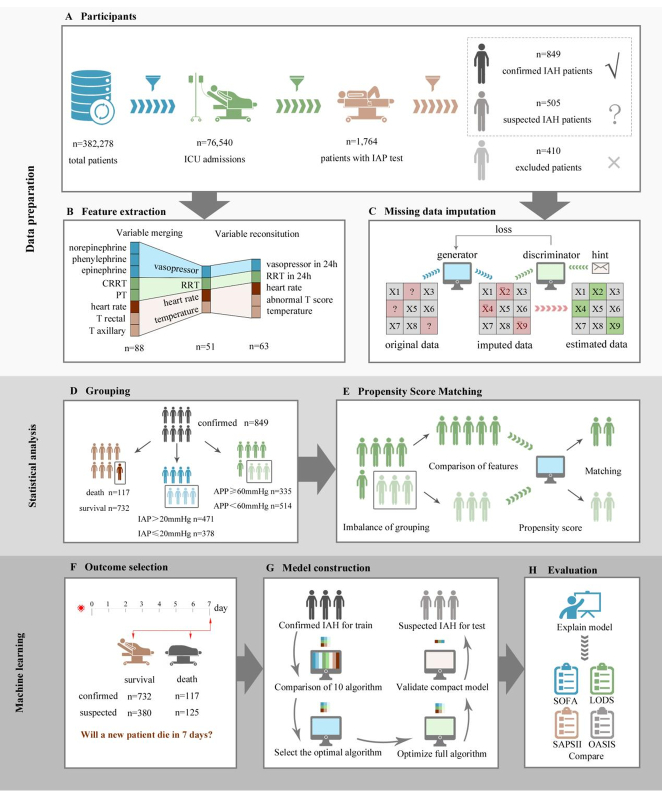

## Introduction

Highlight

- In this study, data of patients with intra-abdominal hypertension were extracted from MIMIC database. Subgroup comparison, propensity matching score grouping, and machine learning were used to investigate key prognostic factors and accurately predict the risk of death.

Intra-abdominal hypertension (IAH) and abdominal compartment syndrome (ACS) are prevalent in ICU patients and cause significant mortality^1^. In 2006, the World Society of Abdominal Compartment Syndrome (WSACS) reached a consensus on IAH and ACS definitions and the technique for measuring intra-abdominal pressure (IAP)^1,2^. Several studies on IAH/ACS and its clinical relevance in ICU patients have been published. ACS directly affects the abdominal hemodynamic environment, causing damage to other compartments and organs^3^. Abdominal perfusion pressure (APP), calculated as the mean arterial pressure (MAP) minus the IAP, has been suggested as the abdominal analogue to cerebral perfusion pressure and a potential endpoint for resuscitation^4^. Although the systemic hemodynamics remain stable when IAH occurs, APP decreases, and visceral ischemia and hypoxic injury may form^5–7^. Therefore, identifying the risk factors contributing to mortality in ICU patients with IAH/ACS and developing a model for predicting mortality are essential to help clinicians with immediate and appropriate decision-making regarding treatment options.

Currently, a large amount of data has been accumulated for ICU patients’ diagnosis and treatment; effectively using this large explosive data is a pressing problem to be solved. The dynamic monitoring of various indicators in ICU patients can be clinically instructive. However, clinical data are often incomplete and inconsistent because of various subjective and objective factors. This problem limits adequate sample size with strong noise for model design, consequently causing difficulties in generalizing the designed prognostic models to other clinical datasets with incomplete data; moreover, it greatly limits the size of the beneficiary patient population. However, with the emergence and rapid development of machine learning (ML) algorithms, it has become possible to accurately analyze such complex multifactors and predict their outcomes^8^.

This study aimed to improve the death risk warning capability for clinical application in patients with IAH and to demonstrate that a high-performance prediction model can be derived from queues with severely missing data. With such a prognostic model, high-risk patients could be identified on time, enabling aggressive, and precise clinical procedures.

## Materials and methods

### Participants

**Figure 1:**
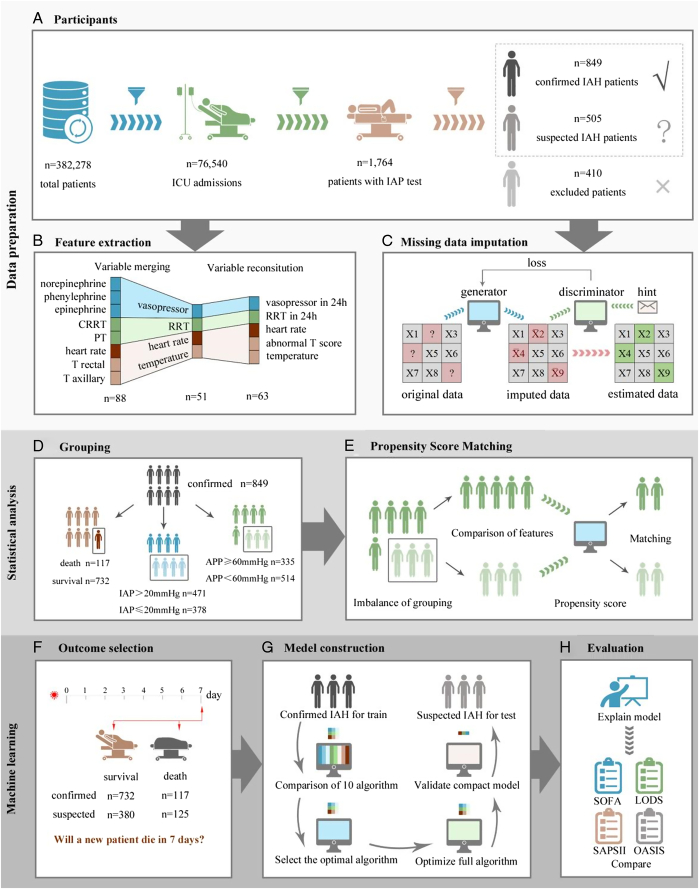
Schematic illustration of the study design. (A) The participants screening process. We first extracted 76 540 adults admitted to the ICU unit from 382 278 documented patients. Only 1764 patients underwent a valid bladder pressure test, where 849 patients were confirmed IAH, 505 were suspected, and 410 were excluded. (B) Feature extraction. We extracted 88 features that were closely related to patient prognosis, and finally 63 key features were obtained through reconstitution. (C) Missing data imputation. The incomplete data was filled using Generative Adversarial Imputation Nets. (D) Grouping. Patients in the confirmed cohort were classified and compared in different ways. (E) Propensity Score Matching. PSM was used to reduce differences in other variables between the groups to observe the effect of APP on survival. (F) Outcome selection. The primary outcome was 7-day mortality because short-term survival is important in clinical decision-making. (G) Model construction. The cohort of confirmed IAH patients were used to train the model, and the best one was selected for optimization. Then, the number of features was compressed to build a compact model, which was tested in suspected patients. (H) Evaluating model. The established model was compared with the commonly used clinical scoring systems, and finally developed as a software. LODS, logistic organ dysfunction score; OASIS, oxford acute severity of illness score; SAPSII, simplified acute physiology score II; SOFA, sequential organ failure assessment.

This was a retrospective study using the Medical Information Mart for Intensive Care IV (MIMIC-IV version 1.0) database between 2008 and 2019^9^. MIMIC-IV is a large, freely available database comprising anonymous health-related data from 382 278 patients admitted to the critical care units of the Beth Israel Deaconess. Information was extracted from all ICU patients aged >18 years and who had undergone effective bladder pressure tests. Due to the absence of IAH diagnostic codes in the International Classification of Diseases (ICD) and the fact that the diagnosis of ACS does not meet our experimental setting, we used the guideline definition as a diagnostic basis to screen patients. Notably, the WSACS diagnostic definition for IAH/ACS emphasizes continuous bladder pressure testing; a patient with only one IAP test of >12 mmHg is a suspect. Jiao He (co-author) was authorized to use the database and was responsible for the information extraction process. The flowchart is shown in Figure 1A. Lastly, patients with >24 h of ICU stay were included in the study.

### Feature extraction

We chose the first day in the ICU as the data acquisition node and worst-case feature for data analysis reflecting the etiology and pathological changes in ACS/IAH during the ICU stay. At the initial stage, 88 features were collected, including demographic characteristics, vital signs, laboratory data, critical clinical interventions, and urine output. Additionally, complications and comorbidities associated with ACS/IAH were collected according to ICD codes. Next, we performed the feature reconstitution on the collected data. Consequently, 51 features were obtained by feature merging; the distribution of the partial data is shown in Table S1 (Supplemental Digital Content 1, http://links.lww.com/JS9/D261). Notably, for some continuous features, the fluctuation intervals of the feature values are more valuable than the primary values. Therefore, we denoted these features with intervals based on professional clinical knowledge. Lastly, 63 characteristic features were obtained (Table S2, Supplemental Digital Content 1, http://links.lww.com/JS9/D261). A simplified flow of the feature engineering is shown in Figure 1B.

### Missing data imputation

To address the missing data problem, we used generative adversarial imputation nets (GAIN) derived from the well-known generative adversarial nets (GAN) to fill in the missing data^10,11^. The basic goal of a GAN network is to find the Nash equilibrium point so that the generator (G) and discriminator (D) loss can achieve the same optimal result. In GAIN, the G observes some components of a real data vector, imputes the missing components conditioned on what is observed, and outputs a completed vector. The D then takes the completed vector and attempts to determine which components were observed and imputed. D is provided additional information as a hint vector to ensure that D forces G to learn the desired distribution. In our study, we first used G to generate missing data according to the complete data distribution. Second, the filled and complete data were used for neural network training. Third, we used D to determine whether the generated and missing data belong to the same class; otherwise, the steps described above were repeated. This basic principle flowchart is shown in Figure 1C. The completeness of the clinical data is shown in Figures S1 A and B (Supplemental Digital Content 1, http://links.lww.com/JS9/D261).

### Statistical analysis

To evaluate the effect of decreased APP on the course of disease during ICU stay, we performed a grouping analysis of the data related to APP. Referring to WSACS, 60 mmHg was used as a threshold for APP value, as it is a distinguishing condition for patients with ACS. Patients with APP ≥60 mmHg were classified as normal APP group, while those with APP <60 mmHg were classified as abnormal APP group. For comparison, patients with 12 ≤ IAP≤ 20, considered IAH Grades I and II, were classified as the moderately increased group, and those with IAP >20 as the severely increased group. This process is illustrated in Figure 1D.

Continuous features with normal distribution were analyzed using an independent sample *t*-test. Distributed features, described as medians, were compared using the Mann–Whitney *U* test. Categorical features, expressed as percentages, were compared using the *χ* ^2^ test. Kaplan–Meier (KM) curves were drawn to evaluate the patient’s survival curve. The differences between groups were compared using log-rank and Breslow tests, and restricted mean survival time analysis was performed to detect the association between the KM curves.

Furthermore, propensity score matching (PSM) was performed to balance patient characteristics between the groups. We used a logistic regression model to calculate the propensity score and match 1:1 for both groups. Next, we used standardized mean differences to evaluate the balance of characteristics after PSM. When the value was >0.2, it was considered imbalanced. PSM was used to analyze the effect of different subgroups of APP on survival after eliminating intergroup differences. This part of the PSM process is shown in Figure 1E. All statistical analyses were performed using SPSS 26.0.0.0 (IBM), and a statistically significant difference was set at *P*<0.05.

### Outcomes

Our primary concern was to predict the patients’ 7-day mortality, as shown in Figure 1F. Secondary outcomes included the impact of decreased APP on patient survival, the mortality of different time frames, and the survival curves of different APP groups.

### Model construction

For model construction, we included patients with confirmed and suspected IAH according to the inclusion criteria mentioned above. As shown in Figure 1G, 10 ML algorithms were preliminarily used to predict 7-day mortality in confirmed patients. Next, accuracy, the area under the receiver operating characteristic curve (AUC), sensitivity, specificity, and the Youden index were calculated to evaluate each model. The algorithm with the largest AUC and Youden index was selected, and the significance of features was assessed using the Shapley Additive Explanations (SHAP) values. Regarding the mean SHAP values, we selected the 20 most essential features to build a compact prediction model using an optimal algorithm. All the ML methods were implemented in Python (version 3.8.3).

### Evaluating model

In the confirmed group, we divided the data by 7:3 into training and testing sets, 594 and 255 cases, respectively. We tested the built model in the suspected group (505 cases) to evaluate its generalizability. The predictive performance of the full and compact models was further compared to that of the commonly used scoring systems^12–15^, as shown in Figure 1H.

## Results

### Baseline characteristics

We screened 1764 adults who underwent bladder pressure tests. Patients hospitalized for <24 h whose bladder pressure tests were all <12 mmHg were excluded. Among the remaining 1354 patients, 849 with two or more bladder pressure values >12 mmHg were the confirmed group, and the remaining 505 were classified as the suspected group. A comparison of the baseline characteristics between the 7-day survival and death groups in the confirmed group is summarized in Table 1. Patients with comorbidities, such as hepatobiliary diseases, tumors, and acid-base imbalance had higher mortality rates. In addition, this table showed that older patients who received emergency interventions had a higher death risk. In the laboratory tests, we found that in the death group, multiple indicators of organ function were more abnormal, such as coagulation dysfunction with an increased international normalized ratio (INR) and prothrombin time (PT), renal dysfunction with decreased urine output and increased creatinine, and liver function damage with increased alanine transaminase and total bilirubin levels.

**Table 1:** Comparison of features of 7-day mortality.

Feature	Survival	Death	*P*
N	732	117	
Demographic feature			
Age	58 (49–69)	63 (52–73)	0.003
Male (%)	469/732 (64.1)	76/117 (65.0)	0.853
Weight (kg)	88.0 (72–100)	87.5 (72–100)	0.854
BMI	30.5 (25.4–34.1)	30.4 (25.6–34.0)	0.959
Tobacco use	120/732 (16.2)	24/117 (20.5)	0.270
Alcohol abuse	190/732 (26.0)	31/117 (26.5)	0.902
Severity of illness			
SOFA	11 (8–14)	14 (11–17)	＜0.001
LODS	9 (6–12)	11 (8–13)	＜0.001
SAPSII score	48 (37–57)	59 (51–67)	＜0.001
OASIS	42 (36–49)	46 (41–53)	＜0.001
GCS score	14 (14–15)	15 (14–15)	0.030
Interventions			
MV use (in 24 h)	433/732 (59.2)	88/117 (75.2)	0.001
RRT use (in 24 h)	59/732 (8.1)	25/117 (21.4)	＜0.001
Vasopressor (in 24 h)	321/732 (43.9)	84/117 (71.8)	＜0.001
Chronic comorbidities			
Hypertension	291/732 (39.8)	42/117 (35.9)	0.428
Diabetes	204/732 (27.9)	32/117 (27.4)	0.907
CHF	130/732 (17.8)	21/117 (17.9)	0.960
COPD	147/732 (20.1)	30/117 (25.6)	0.169
Malignant neoplasm	80/732 (10.9)	22/117 (18.8)	0.015
Leukemia/lymphoma	36/732 (4.9)	2/117 (1.7)	0.119
Hepatobiliary disease	390/732 (53.3)	78/117 (66.7)	0.007
Chronic renal disease	137/732 (18.7)	27/117 (23.1)	0.267
Acute comorbidities			
Stroke	51/732 (7.0)	5/117 (4.3)	0.276
Sepsis/septicemia	372/732 (50.8)	79/117 (67.5)	0.001
Acute pancreatitis	122/732 (16.7)	10/117 (8.5)	0.024
Acid-based unbalance	391/732 (53.4)	86/117 (73.5)	＜0.001
CHD	114/732 (15.6)	19/117 (16.2)	0.854
Ascites	204/732 (27.9)	32/117 (27.4)	0.907
Vital signs			
APP (mmHg)	58 (50–66)	53 (46–60)	＜0.001
CVP (mmHg)	16 (9–17)	14 (9–20)	0.667
Heart rate (bpm)	97 (83–110)	97 (84–111)	0.864
Temperature (°C)	36.9 (36.6–37.3)	36.4 (35.9–37.0)	＜0.001
Respiratory rate (bpm)	21 (17.6–23.9)	23 (18–26)	＜0.001
Urine output (ml)	1258 (500–1702)	691 (100–779)	＜0.001
Laboratory tests			
WBC (10^9^/l)	16.3 (9.9–20.8)	20.3 (11.1–23.6)	0.011
Platelet count (10^9^/l)	213 (122–269)	193 (103–246)	0.129
Hemoglobin (10^12^/l)	11.5 (9.9–13.0)	11.7 (10.1–13.1)	0.461
Creatinine (mg/dl)	2.2 (1.1–2.7)	3.0 (1.8–3.6)	0.024
BUN (mg/dl)	37 (19–46)	47 (24–58)	0.001
Albumin (g/dl)	2.9 (2.4–3.4)	2.9 (2.4–3.4)	0.794
ALT (IU/l)	37 (21–115)	93 (34–463)	0.002
AST (IU/l)	72 (37–238)	229 (83–1213)	0.002
Total bilirubin (mg/dl)	4.6 (0.7–4.5)	6.8 (1.2–8.5)	0.013
INR	2.0 (1.3–2.2)	2.8 (1.8–3.3)	＜0.001
PT (sec)	21.3 (14.6–23.4)	29.8 (19.7–34.7)	＜0.001
Fibrinogen (mg/dl)	314 (180–405)	229 (130–247)	＜0.001
CK-MB (ng/ml)	7 (3–19)	12 (4–29)	0.949
Glucose (mg/ml)	192 (127–217)	212 (128–255)	0.099
Calcium (mg/dl)	7.5 (6.9–8.1)	7.4 (6.8–8.0)	0.289
PCO_2_ (mmHg)	49 (41–56)	51 (40–56)	0.309
PaO_2_/FiO_2_ ratio	154 (85–200)	123 (79–156)	＜0.001
Potassium (mmol/l)	4.2 (3.6–4.7)	4.4 (3.7–4.9)	0.021
Sodium (mmol/l)	136 (133–139)	135 (131–139)	0.051

BUN, blood urea nitrogen; CHD, coronary heart disease; CHF congestive heart failure; COPD, chronic obstructive pulmonary disease; FiO_2_, fraction of inspiration oxygen; MV, mechanical ventilation; PaO_2_, arterial partial pressure of oxygen; PCO_2_ partial pressure of carbon dioxide; RRT, Renal replacement therapy.

### Incomplete data imputation

We used the GAIN method for data imputation and compared it with other commonly used algorithms, including mean, zero-value, and K nearest neighbor fillers. The root mean square error (RMSE) and median absolute deviation (MAD) were used to evaluate the different data imputation methods, where a small value indicates better performance. In the experiment, all the complete data from the confirmed and suspected groups were used to train the models, and the trained models were further used in incomplete data imputation. Notably, the RMSE penalty for outliers was larger than that of MAD. As shown in Figure S1 C and D (Supplemental Digital Content 1, http://links.lww.com/JS9/D261), GAIN achieved the best performance in MAD (0.200) and RSME (0.345) and was used in this study.

### The role of APP in survival

Comparing the different IAP collection time points and survival times, the APP value was the best indicator, as the mean difference between survival and death group was statistically significant. The difference in mean APP values was also most obvious in the 2–7-day death and survival groups (Table S3, Supplemental Digital Content 1, http://links.lww.com/JS9/D261).

**Figure 2:**
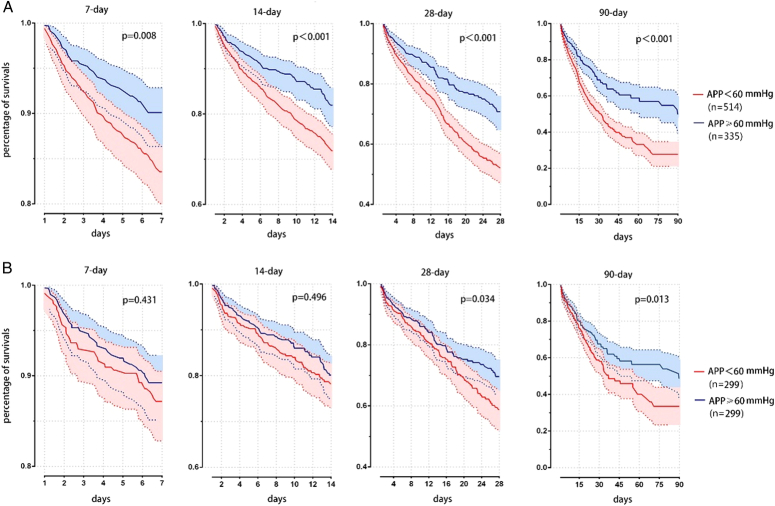
Survival comparison of APP level between the original and the PSM cohort; (A) Survival curves of the original cohort; (B) Survival curves of the PSM cohort.

The mortality of the severely increased IAP group was significantly higher than that of the moderately increased IAP group at 7 (15.9% vs. 11.1%, *P*=0.043) and 90 (44.6% vs. 36.8%, *P*=0.021) days. Patients with an abnormal APP <60 mmHg had significantly higher all-cause mortality within 7 days to 90 days compared to patients who had a normal APP of more than 60 mmHg. The increase in mortality accumulated over time, and by day 90 the difference between the two groups had reached 20% of the total (49.0% vs. 29.0%, *P*<0.001). The comparison of mortality differences is shown in Table S4 (Supplemental Digital Content 1, http://links.lww.com/JS9/D261). The difference remained significant in long-term survival at 28 (33.4% vs. 25.4%, *P*=0.034) and 90 (40.1% vs. 30.8%, *P*=0.013) days even after PSM eliminated other mortality risks between the groups. The baseline characteristics of the APP cohort before and after PSM are shown in Table S5 (Supplemental Digital Content 1, http://links.lww.com/JS9/D261), and the survival curves are shown in Figure 2. In the density diagram of Fig. S2 (Supplemental Digital Content 1, http://links.lww.com/JS9/D261) an apparent bias distribution of APP can be found.

### 7-day Mortality Prediction

**Figure 3:**
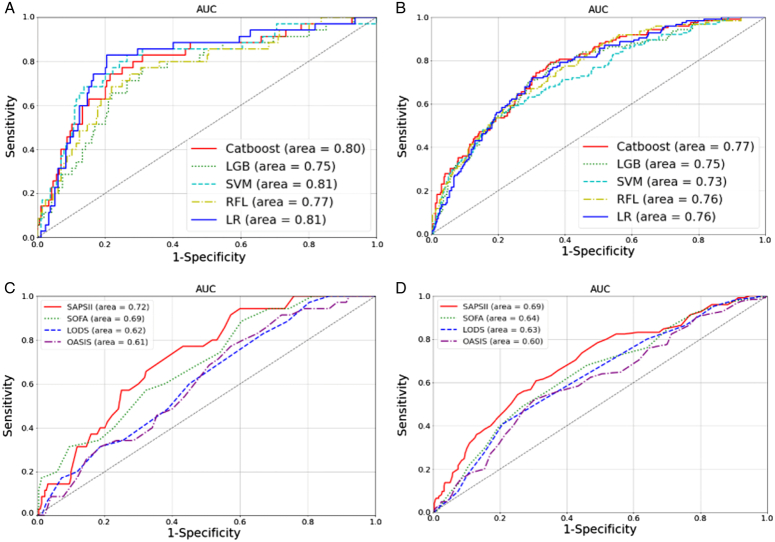
ROC curve of new building model and traditional score systems. (A) The test subset of the confirmed group in multiple machine learning models. (B) The test subset of suspected group in multiple machine learning models. (C) The test subset of the confirmed group in multiple clinical score systems. (D) The test subset of suspected group in multiple clinical score systems.

Furthermore, we built the 7-day mortality prediction models with APP using 10 different ML algorithms. First, the built models were tested on the confirmed IAH group (test set). Next, we tested this model in a suspected IAH group to validate its generalizability. The top five algorithms, namely, Categorical Boosting (CatBoost)^16^, Light Gradient Boosting, Support Vector Machine, Random Forest Classifier, and Logistic Regression, are shown in Figure 3A and B, and the AUC and accuracy are shown in Table S6 (Supplemental Digital Content 1, http://links.lww.com/JS9/D261). Regarding AUC, CatBoost and Logistic Regression obtained the best results, considering their performance in the confirmed and suspected IAH groups. CatBoost had better generalizability in the suspected group; hence, we chose it in the follow-up research and compression model construction. Notably, all the top five ML algorithms obtained an AUC >0.75 using APP, demonstrating its importance in modality prediction.

**Figure 4:**
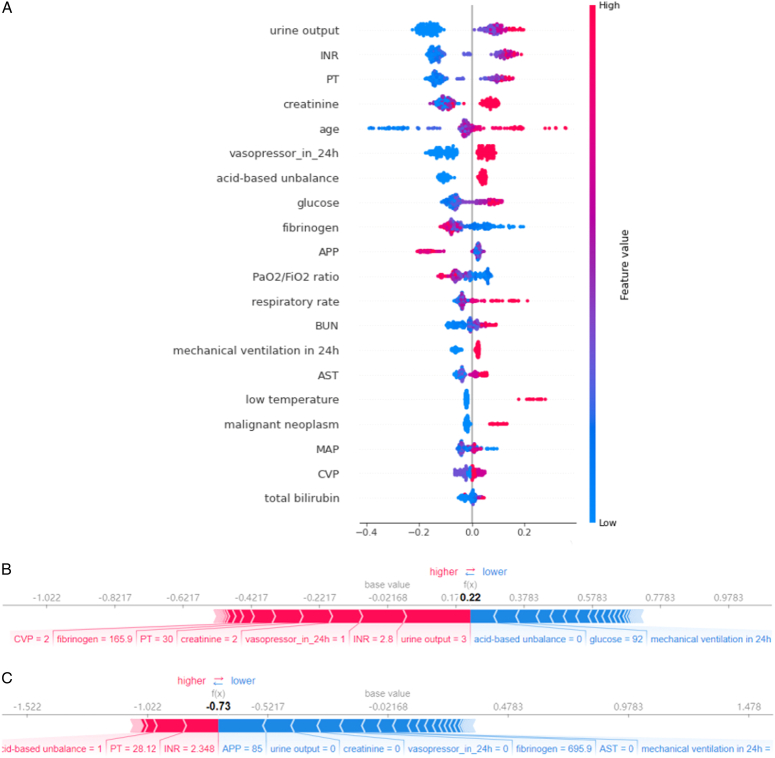
Model visualization analysis. (A) SHAP values of the Cat Boost Model (B) One patient who was diagnosed as low-risk and survived. (C) One patient who was diagnosed as high-risk and died.

After choosing CatBoost as the final model, we built a compact model using SHAP values based on the significance analysis in the prediction results^17^. The summary chart ranks the features according to the sum of the SHAP values of all samples and shows the distribution of the influence of each feature on the entire model (Fig. 4A). The compact model composed of these top 20 features by SHAP ranking had a slightly smaller AUC in both groups; however, it is more convenient in clinical application.

### Model interpretation and clinical score comparison

As shown in Figure 4A, urine output, INR, and PT were the most important features for predicting 7-day mortality. The selected indicators included coagulation function (INR, PT, and fibrinogen), renal function (urine output, creatinine, and blood urea nitrogen), basic clinical history (age, malignancy, temperature, and respiratory rate), interventions (early application of vasopressors and mechanical ventilation), metabolic functions (acid-base imbalance and glucose), liver function (AST and total bilirubin), blood circulation system (APP, MAP, and CVP), and the respiratory system (PaO_2_/FiO_2_ ratio).

The prediction results of these two specific instances are shown in Figure 4. The bars in red and blue represent risk and protective factors, respectively, and longer bars represent greater feature importance. As shown in Figure 4B, the patient had poor urine output with a high INR time and early use of vasopressors, our model successfully predicted that she would die within 7 days. Moreover, as shown in Figure 4C, the patient had a relatively mild condition; the model predicted a low-risk value, and the patient survived for >7 days.

Furthermore, considering the commonly used clinical scoring system, there were no guiding 7-day mortality reference data; hence the receiver operating characteristic curves of the two cohorts were first drawn, as shown in Figure 3 C and D. The threshold points were then determined according to the best Youden index of the confirmed cohort, and the suspected cohort was used for further verification. In the traditional clinical scoring system, SAPSII had the highest efficacy in predicting IAH mortality, with an AUC of 0.72 in the confirmed cohort and 0.69 in the suspected cohort. However, the Youden index of SAPSII was only 0.34 and 0.17 in confirmed and suspected patients, respectively. The full model we built achieved the best results with AUC 0.80 in the confirmed cohort and 0.77 in the suspected cohorts. The specific performance parameters of the new models and the traditional scoring systems are listed in Table 2.

**Table 2:** Comparison of performance between traditional clinical score and new model.

	Confirmed IAH validation				Suspected IAH validation			
Model	AUC	Youden	Sensitivity	Specificity	AUC	Youden	Sensitivity	Specificity
The full model	**0.80 [0.75–0.86]**	**0.53**	0.74 [0.58–0.86]	**0.79 [0.73**–**0.84]**	**0.77 [0.74**–**0.81]**	0.33	0.56 [0.50–0.67]	**0.77 [0.72**–**0.81]**
The compact model	0.78 [0.73–0.82]	0.52	0.83 [0.64–0.90]	0.69 [0.62–0.74]	0.76 [0.73–0.79]	**0.41**	0.73 [0.64–0.80]	0.68 [0.63–0.72]
SOFA score	0.69 [0.60–0.78]	0.29	0.89 [0.72–0.96]	0.40 [0.33–0.46]	0.64 [0.59–0.70]	0.17	0.73 [0.64–0.80]	0.44 [0.39–0.49]
LODS score	0.62 [0.52–0.71]	0.17	**0.97 [0.83**–**0.99]**	0.20 [0.15–0.26]	0.63 [0.57–0.68]	0.11	**0.95 [0.89**–**0.98]**	0.16 [0.13–0.20]
SAPSII score	0.72 [0.64–0.80]	0.34	0.94 [0.79–0.99]	0.40 [0.34–0.47]	0.69 [0.64–0.75]	0.17	0.83 [0.75–0.89]	0.34 [0.29–0.39]
OASIS score	0.61 [0.52–0.70]	0.20	0.77 [0.59–0.89]	0.43 [0.36–0.50]	0.61 [0.55–0.66]	0.15	0.62 [0.53–0.71]	0.53 [0.48–0.58]

Best performance was identified with bold.

## Discussion

APP reduction is a significant predictor of survival, affecting the survival prognosis of patients with IAH. After strictly excluding all potential risks of death, APP remained as an independent long-term death risk. APP is more robust than IAP and MAP in the assessment of survival in patients with IAH. We constructed a robust model to predict the 7-day mortality probability of patients with IAH, which is superior to the commonly used clinical scoring systems. Meanwhile, we also obtained a ranking of the risk factors strongly associated with death in these patients. We believe this is the first survival prognosis model based on a large database for patients with IAH.

MIMIC-IV is one of the best-known open ICU databases in the world; however, only 1764 of the 76 540 admitted ICU patients were tested for IAP^9^. Many patients with high-risk factors for IAH were not tested for IAP. Furthermore, we found that up to 40% of the patients (706 out of 1764) in this database did not perform more than twice the IAP tests as required by the WSACS guidelines^2^. Additionally, many enrolled patients did not have routine and important clinical data. Missing data are a common phenomenon in clinical work; however, inferring results from existing data is still of great value. According to the data collection and screening of patients with IAH, 88 features closely related to the prognosis of patients were extracted, and 63 indices were finally obtained through merging and reconstitution. These features had some missing; therefore, our work was to find a way to fill in the missing data and maximize the authenticity and reliability of data filling. Medical researchers are using machine learning algorithms to fill in the missing data, which has the advantages of being closer, more robust, and more balanced with real data. Compared with traditional filling methods, such as mean filling, machine learning’s GAIN filling method adds a hint layer, balances and evaluates relevant clinical indicators, and maximizes the authenticity and reliability of data filling. We achieved the best performance (MAD 0.200 and RMSE 0.345) using the GAIN data imputation method, indicating that this approach is desirable. In addition, we also used SHAP, an advanced AI interpretation technology, to change the black box state of the machine algorithm into a state understandable by human logical thinking. Combined with an unprecedented large sample size and multiple disease groups, the model in this study is more generalized and can be expanded to more application conditions.

IAH is highly prevalent among all types of patients admitted to the ICU, with an incidence of about 35%^18^. Once ACS develops in these patients, the mortality rate is as high as 38–71%^19^. Therefore, the advanced prediction of IAH-high-risk patients is of great clinical significance. In previous studies and clinical practice, the IAP is the primary basis for prediction^1,2,20^. However, IAP prediction has the following problems: first, patients with obesity do not apply to the standard of 12 mmHg^21,22^; second, IAP varies greatly in different primary diseases^22^; thirdly, IAP is the main factor affecting the blood supply to the abdominal organs, a slight increase in IAP can still cause subclinical organ damage^5–7^. At last, IAP value does not directly reflect the perfusion pressure of the abdominal organs^20,23^. Furthermore, WSACS has repeatedly mentioned the importance of APP, APP <60 mmHg has been regarded by some studies as a key indicator of new organ dysfunction^5,24^. Not surprisingly, in our study, the cohorts divided by APP 60 mmHg had significantly stronger differences in mortality than those divided by IAP 20 mmHg, regarding statistical results and model construction. We believe that APP is a better indicator because it combines information from IAP and MAP, narrowing down the range of normal thresholds. Therefore, decreased APP can more directly reflect the state of compensatory failure of the somatic function.

Adequate APP guarantees abdominal organ function, which depends on arterial blood flow, venous return flow, and the response of the abdominal space to an increase in intestinal contents^4^. Consequently, APP deficiency causes hypoxic injury to abdominal organs^5^. The gastrointestinal tract is the trigger organ for multiple organ dysfunction syndromes in clinically critical patients and is most affected by APP^25^. In animal experiments, it was observed that with a decrease in APP, intestinal blood perfusion decreased, intestinal mucosa showed ischemic and hypoxic damage, intestinal mucosal barrier function was lost, and intestinal infections occurred^26^. Furthermore, the infection promotes bacteria and endotoxins in the intestine to continuously enter the blood circulation, creating a vicious cycle^5,25^. Simultaneously, renal function was significantly affected by the decrease in APP. When APP was <60 mmHg, the kidney was hypoperfused, the glomerular filtration rate decreased, and the structure and function of renal tubular epithelial cells were damaged^27^. As in our prediction model, renal function plays an important role, of which urine output, creatinine and BUN are all important predictors. Additionally, insufficient APP can significantly reduce hepatic blood flow and significantly increase vascular resistance, resulting in varying degrees of damage to liver function. In the presence of primary liver lesions, hypoperfusion caused by decreased APP levels has a particularly prominent effect on liver function^28,29^. The importance of liver function impairment to death was also demonstrated in our model, and associated coagulation disorders (INR, PT, and fibrinogen) and increased damage factors (AST and total bilirubin) were also critical features. Moreover, decreased APP can cause glucose metabolism and mitochondrial function dysfunction, inhibit peritoneal and lymphatic fluid absorption, and reduce blood supply to other abdominal organs, such as the adrenal gland and pancreas^30–32^. When it comes to metabolic disorders, the effects of acid-base imbalance and glucose in our model should not be underestimated. Lastly, the continuous decline of APP directly affects intracranial pressure and can have a deleterious impact on patients with a wide variety of neurological pathologies, from traumatic brain injury to intracranial hypertension^33^. Therefore, patients with IAH are more likely to develop multiple organ dysfunction syndrome, which can cause fatal blows^5,24,26^.

Furthermore, in clinical practice, the use of APP is very convenient; its calculation is simple and noninvasive, making it safer than CVP or left atrial pressure. Some previous studies have confirmed that the commonly used CVP and MAP are unreliable hemodynamic parameters for fluid resuscitation in patients with elevated IAP^20,34^. Since patients with IAH and ACS have significant intestinal wall edema and an increase in peritoneal contents, it is insufficient to expect improvement by simply restoring MAP to normal by infusion^34,35^. Existing studies have used APP as the resuscitation endpoint and survival predictor in patients with IAH and confirmed that APP is superior to other indicators, such as IAP, blood pH, buffer base, urine output volume, and MAP^4^. Other studies have proposed using APP instead of IAP and MAP as evaluation parameters for hemodynamic changes in the internal organs of patients with IAH patients^36^. Furthermore, active blood volume restoration, dopamine, and diuretics had no significant effect on the renal function of these patients; therefore, IAP should be reduced to a normal level after adequate drainage of abdominal fluid and open decompression^37^. In other words, the above process gradually restores the APP to the physiological level according to the comprehensive consideration of the patient’s systemic and local conditions. However, in current clinical practice, APP detection in critically ill patients, such as those with shock, has not attracted sufficient attention. From the results of this study, although APP is a major determinant of critically ill patient outcomes, a single threshold for APP cannot be applied globally to decision-making in all critically ill patients, as it oversimplifies the complex physiological processes.

We conducted feature-engineering processing after the original data collection to improve the prediction model’s performance. Feature engineering is the selection process of data representation that involves processing the original data to increase the recognition and specificity of the data by machine learning^38^. For example, in the region of normal body temperature, extremely high and low body temperatures will have a certain impact on the life state. Therefore, we combined the relevant professional knowledge and scoring systems to assign them values in rank features and integrated the new features into the original data source. Notably, common clinical scores, such as SOFA, LODS, SAPSII, and OASIS, were excluded from the prediction model. These scoring methods showed strong clinical application and were correlated with 7-day mortality. However, we chose to exclude them mainly because we had covered almost all feature selection characteristics and because it is inherently inconvenient to calculate multiple scores in clinical applications. Similar to our model, these scoring systems rely on collecting relevant clinical features. Feature selection is typically performed by converting one or two indicators into scores representing a biological system. The primary purpose of these scores is to make a preliminary judgment of the disease severity, and there are no specific reference criteria for prognosis and outcome.

The mortality prediction model we trained has a wide range of clinical applications. The AI-based filling function can be effectively applied in situations such as when an unconscious patient cannot provide a history, when test results are incomplete in an emergency, or when contraindications exist for certain tests. Explainable AI functions can assist clinicians in managing patients based on the ranking of risk factors, helping to provide comprehensive care. Our team has trained multiple models of different diseases through multidisciplinary collaboration^39,40^. We are preparing to integrate and generalize these models and modular assembly to build a new kind of ward system with functions such as automated data aggregation, disease assessment, clinical decision recommendations, and model retraining. Our study has some limitations that can be addressed. First, we conducted a retrospective study based on a public database, which had many missing data and critical items, such as MMP, N-terminal peptide, and HMGB1, the molecular mediators proposed in recent studies^41–43^. Second, the study had no blank control group of patients with normal IAP because IAP testing is not routine in the ICU. Lastly, the life span of patients collected in the MIMIC-IV database is relatively large, and the mortality rate as an endpoint of clinical studies may vary with advances in medical science.

## Conclusions

Statistical analysis of all 1354 patients in MIMIC-IV database with IAH showed that decreased APP is a key risk factor for the prognosis of death, and its role is more clinically instructive than IAP. The results of the cohort analysis show that APP, as a risk factor for death, continues to accumulate over time and became an important independent risk factor at 28 and 90 days. The 7-day mortality prediction model of patients with IAH constructed by us has better performance than all current scoring systems, which can provide an important basis for guiding clinical decision-making and effectively help medical staff to adjust programs and allocate resources.

## Ethical approval

This database was created with the agreement of the Massachusetts Institute of Technology (Cambridge, MA) and Beth Israel Deaconess Medical Center (Boston, MA), and we have obtained the relevant permission at the time of data collection. This study submitted the relevant research process to Medical Ethics Committee of Second Affiliated Hospital of Army Medical University, PLA, numbered 2023-research No 018-01. After review by the Committee, it is determined that the project meets the conditions for exemption from review.

## Consent

This study is a public database study, and the patient information involved in this study has obtained corresponding permission in the database information collection stage.

## Source of funding

The present study was supported by the National Natural Science Foundation of China (No. 62076247, No. 61701506 to Jingjing Xiao) and the National Key R&D Program of China (No. 2021ZD0140408 to Yan Zhuang).

## Author contribution

L.X.: conceptualized the research aims, contributed to the design of the study, statistical analysis, and model design; W.Z.: contributed to the design of the study, code editing, and model training; J.H.2: contributed to the experimental design, extracting the data, and interpreting the model; J.H.4 and S.H.: contributed to data arrangement and model testing; L.X. and W.Z.: completed the initial manuscript; H.Y.: made critical indications and revisions to the structure and design of the manuscript; Y.Q. and J.X.: supervised the entire research process, providing critical revisions to the manuscript from clinical practice, and machine learning algorithm, respectively. All authors read and approve the final manuscript.

## Conflicts of interest disclosure

The authors declare that they have no financial conflict of interest with regard to the content of this report.

## Research registration unique identifying number (UIN)

This was a database-based study, and no humans were involved in the experimental diagnosis or treatment.

## Guarantor

Yuan Qiu, Department of General Surgery, The Second Affiliated Hospital of the Army Medical University, Chongqing 400037, People’s Republic of China. Jingjing Xiao, Department of Medical Engineering, The Second Affiliated Hospital of the Army Medical University, Chongqing 400037, People’s Republic of China.

## Data availability statement

The datasets provided in this study can be obtained from the MIMIC-IV official website (https://physionet.org/content/mimiciv/1.0/).

## Provenance and peer review

No, this paper is not invited.

## Assistance with the study

None.

## Presentation

None.

## Database usage statement

The information involved in this study was created with the agreement of Massachusetts Institute of Technology (Cambridge, MA) and Beth Israel Deaconess Medical Center (Boston, MA), and one of our authors (Jiao He) has obtained the relevant permission at the time of data collection. Therefore, the ethical approval statement and informed consent were not submitted in this manuscript.·Background information on the database can be accessed at the following website: https://physionet.org/about/licenses/open-data-commons-attribution-license-v10/·The clinical data covered by the database can be downloaded from the following website: https://physionet.org/content/mimiciv/1.0/ Our data use agreement for MIMIC-IV:

## Supplementary Material
