# Supplementary material for: Abdominal perfusion pressure is critical for survival analysis in patients with intra-abdominal hypertension: mortality prediction using incomplete data
Source: Int J Surg. 2024 Aug 14;111(1):371–81. doi: 10.1097/JS9.0000000000002026 (PMC11745648; doi:10.1097/JS9.0000000000002026)
Supplement: Supplementary file 1 [file js9-111-0371-s001.docx]

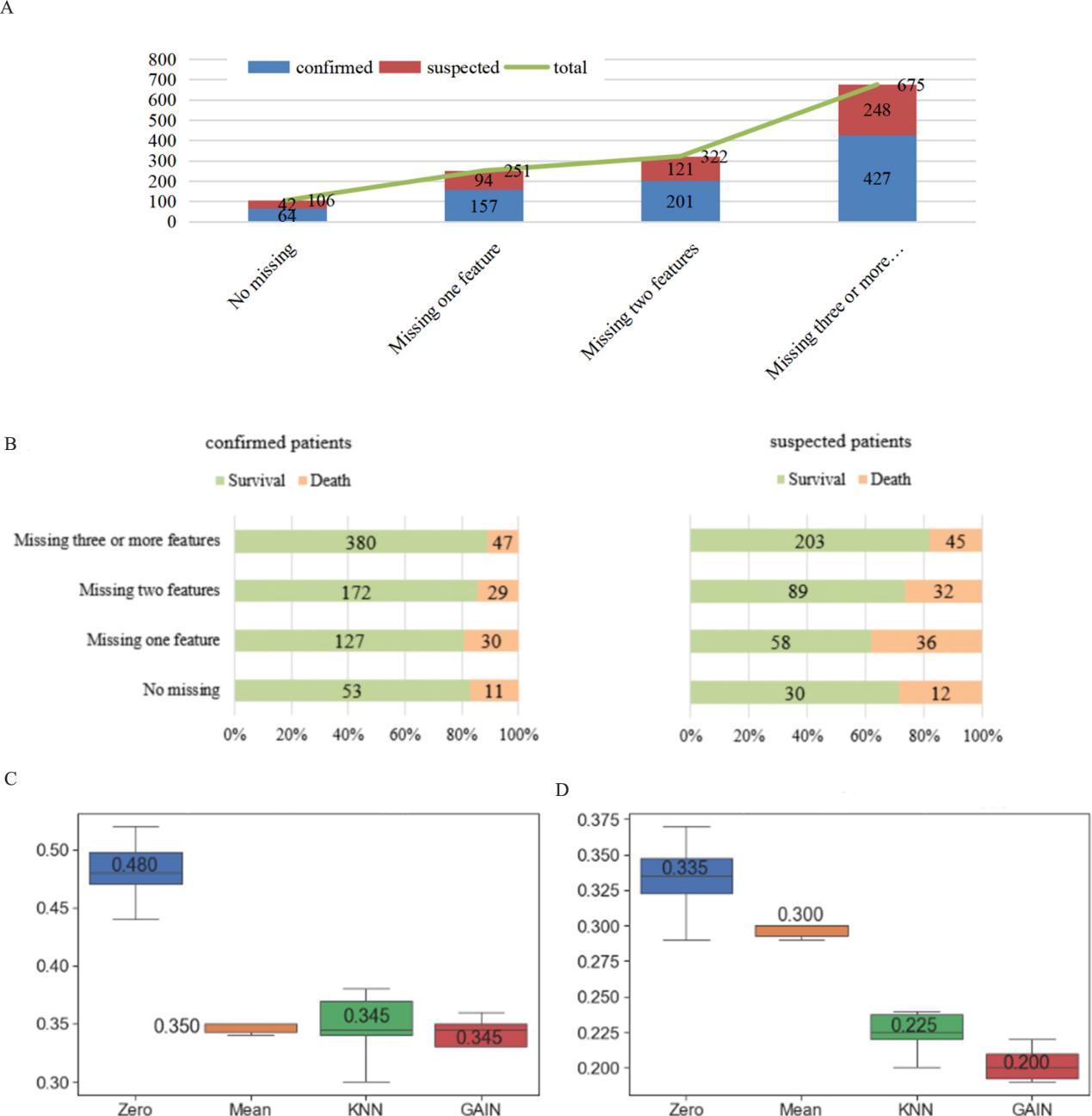


**Figure S1** Clinical data missing status and filling. **A.** Missing data distribution analysis. **B.** Distribution of survival and death among different groups. **C.** RMSE of four filling methods. **D.** MAD of four filling methods.


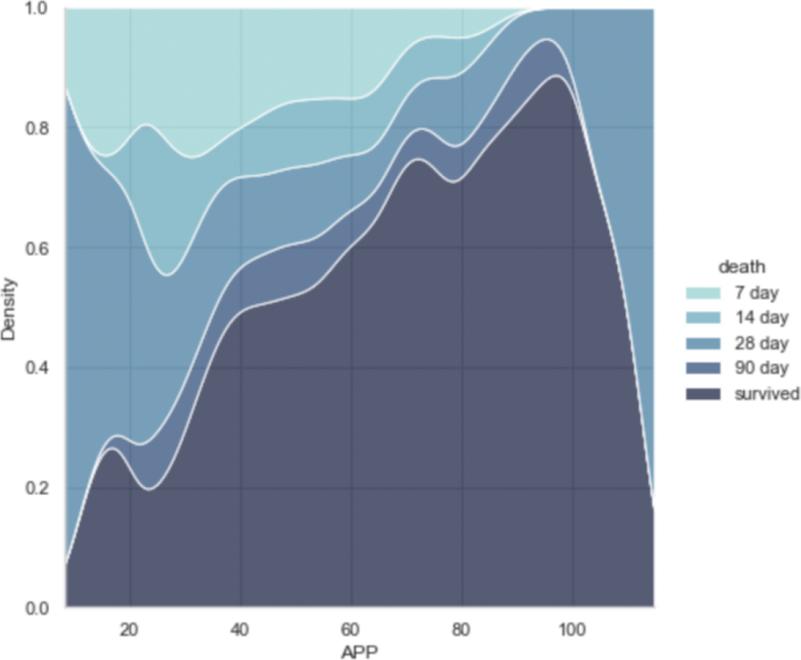


**Figure S2** APP density picture of patients at different time points of death

| **Feature name** |  |  |
| --- | --- | --- |
| **Gender** |  |  |
| Male | 64.19% | 545 admissions |
| Female | 35.81% | 304 admissions |
| **Age** |  |  |
| Median (Mean) | 59(58.86) |  |
| Range | 19-91 |  |
| **Length of stay (days)** |  |  |
| Median (Mean) | 9(13) |  |
| Range | 1-79 |  |
| **ICU types** |  |  |
| SICU | 30.74% | 261 admissions |
| MICU/SICU | 21.67% | 184 admissions |
| MICU | 32.63% | 277 admissions |
| CCU | 14.96% | 127 admissions |
| **Severity of illness** |  |  |
| **Emergency patient score** |  |  |
| SAPSII | 49(49) |  |
| OASIS | 44(43) |  |
| **Multiple organ dysfunction patient score** |  |  |
| SOFA | 12(12) |  |
| LODS | 9(9) |  |
| **Mortality** |  |  |
| 2-day | 3.89% | 33 admissions |
| 7-day | 13.78% | 117 admissions |
| 14-day | 23.09% | 196 admissions |
| 28-day | 34.39% | 292 admissions |
| 90-day | 41.11% | 349 admissions |

**Table S1** Patient characteristics. **Abbreviation:** *SICU* surgical intensive care unit, *MICU* medical intensive care unit, *CCU* cardiac care unit, *SAPSII* simplified acute physiology score ii, *OASIS* oxford acute severity of illness score, *SOFA* sequential organ failure assessment, *LODS* logistic organ dysfunction score.

| **Original feature** | **Value assignment** | | | | |
| --- | --- | --- | --- | --- | --- |
|  | **0** | **1** | **2** | **3** | **4** |
| **CVP** | 5-9 mmHg | 10-12 mmHg | 13-15 mmHg | ≥16 mmHg |  |
|  |  | 3-4 mmHg | 1-2 mmHg | ＜1 mmHg |  |
| **Temperature** | 35.5-37.5 ℃ | ≥37.6 ℃ |  |  |  |
|  |  | ≤35.4 ℃ |  |  |  |
| **WBC** | 4-10 ×10^9^/L | 10.1-15 ×10^9^/L | 15.1-20 ×10^9^/L | 20.1-30 ×10^9^/L | ≥30.1 ×10^9^/L |
|  |  | 3-3.9 ×10^9^/L | 2-2.9 ×10^9^/L | 1-1.9 ×10^9^/L | ＜1 ×10^9^/L |
| **Platelets** | ＞150 ×10^9^/L | 100-150 ×10^9^/L | 50-99 ×10^9^/L | ＜50 ×10^9^/L |  |
| **Urine output** | ＞800 ml | 400-799 ml | 100-399 ml | ＜100 ml |  |
| **Creatinine** | ＜1.2 mg/dL | 1.2-1.9 mg/dL | 2-5.9 mg/dL | ＞5.9 mg/dL |  |
| **ALT** | ≤40 U/L | 41-120 U/L | 121-400 U/L | ＞400 U/L |  |
| **AST** | ≤40 U/L | 41-120 U/L | 121-400 U/L | ＞400 U/L |  |
| **CK-MB** | ＜5 ng/ml | ≥ 5 ng/ml |  |  |  |
| **Potassium** | 3-5.5 mmol/L | ＞5.5 mmol/L |  |  |  |
|  |  | ＜ 3 mmol/L |  |  |  |
| **Sodium** | 130-150 mmol/L | ＞150 mmol/L |  |  |  |
|  |  | ＜130 mmol/L |  |  |  |
| **GCS score** | 15 | 13-14 | 10-12 | 6-9 | ＜6 |

**Table S2** The new features assigned by feature engineering. **Abbreviation:** *CVP* central venous pressure, *WBC* white blood cell count, *ALT* alanine transaminase, *AST* aspartate transaminase, *CK-MB* creatine kinase isoenzyme MB type, *GCS* Glasgow coma scale.

| **Time point** | **1–2-day mortality** | | **2–7-day mortality** | | **7–14-day mortality** | | **14–28-day mortality** | | **28–90-day mortality** | |
| --- | --- | --- | --- | --- | --- | --- | --- | --- | --- | --- |
|  | **Primary cohort** | **p-value** | **Primary cohort** | **p-value** | **Primary cohort** | **p-value** | **Primary cohort** | **p-value** | **Primary cohort** | **p-value** |
| N (survival/dead) | 816/33 | | 732/84 | | 653/79 | | 557/96 | | 500/57 | |
| Minimum IAP | 12.6/16.2 | <0.001 | 12.5/13.9 | 0.016 | 12.5/12.1 | 0.467 | 12.3/13.8 | 0.013 | 12.4/11.5 | 0.169 |
| Maximum IAP | 23.2/25.4 | 0.166 | 23.0/24.9 | 0.072 | 22.9/23.5 | 0.569 | 22.5/25.6 | 0.022 | 22.3/24.0 | 0.072 |
| Initial IAP | 18.7/20.3 | 0.224 | 18.6/19.4 | 0.341 | 18.7/17.9 | 0.393 | 18.3/21.3 | 0.022 | 18.2/19.2 | 0.238 |
| Initial APP | 57.5/52.2 | 0.025 | 58.0/53.2 | 0.001 | 58.3/55.0 | 0.039 | 59.2/53.3 | <0.001 | 59.6/55.5 | 0.023 |

**Table S3** The correlation between different IAP measures in mortality in the primary

|  | **IAP** | | | **APP** | | |
| --- | --- | --- | --- | --- | --- | --- |
|  | **IAP≤20** | **IAP＞20** | **p-value** | **APP≥60** | **APP＜60** | **p-value** |
| N | 378 | 471 |  | 335 | 514 |  |
| 2-day mortality | 10/378(2.6) | 23/471(4.9) | 0.094 | 9/335(2.7) | 24/514(4.7) | 0.144 |
| 7-day mortality | 42/378(11.1) | 75/471(15.9) | 0.043 | 33/335(9.9) | 84/514(16.3) | 0.007 |
| 14-day mortality | 82/378(21.7) | 114/471(24.2) | 0.388 | 57/335(17.0) | 139/514(27.0) | 0.001 |
| 28-day mortality | 117/378(31.0) | 175/471(37.2) | 0.059 | 80/335(23.9) | 212/514(41.2) | ＜0.001 |
| 90-day mortality | 139/378(36.8) | 210/471(44.6) | 0.021 | 97/335(29.0) | 252/514(49.0) | ＜0.001 |

**Table S4** The comparison of different IAP and APP groups on survival time.

| **Feature** | **Original cohort** | | |  | | **PSM cohort** | | | |  |
| --- | --- | --- | --- | --- | --- | --- | --- | --- | --- | --- |
|  | **APP≥60** | **APP＜60** | **SMD** | |  | | **APP≥60** | **APP＜60** | **SMD** | |
| **N** | 335 | 514 |  | |  | | 299 | 299 |  | |
| **Demographic feature** |  |  |  | |  | |  |  |  | |
| Age | 57(47-68) | 60(50-71) | 0.157^*^ | |  | | 58(48-69) | 59(49-73) | 0.081 | |
| Male (%) | 210/335(62.7) | 335/514(65.2) | 0.051 | |  | | 189/299 (63.2) | 189/299 (63.2) | <0.001 | |
| Weight(kg) | 84.2(70.2-95.3) | 90.4(72.8-103.9) | 0.275^***^ | |  | | 84.6(70.0-96.8) | 85.8(70.0-99.0) | 0.053 | |
| BMI | 29.0(24.7-31.5) | 31.1(25.6-35.6) | 0.298^***^ | |  | | 29.1(24.7-31.6) | 29.7(24.6-33.4) | 0.073 | |
| Tobacco use | 50/335(14.9) | 94/514(18.3) | 0.094 | |  | | 44/299 (14.7) | 46/299 (15.4) | 0.019 | |
| Alcohol abuse | 84/335(25.1) | 137/514(26.7) | 0.036 | |  | | 74/299 (24.7) | 74/299 (24.7) | <0.001 | |
| **Interventions** |  |  |  | |  | |  |  |  | |
| MV use (in 24h) | 209/335(62.4) | 312/514(60.7) | 0.035 | |  | | 186/299 (62.2) | 177/299 (59.2) | 0.062 | |
| RRT use (in 24h) | 21/335(6.3) | 63/514(12.3) | 0.247^**^ | |  | | 21/299 (7.0) | 23/299 (7.7) | 0.028 | |
| Vasopressor (in 24h) | 123/335(36.7) | 282/514(54.9) | 0.376^***^ | |  | | 119/299 (39.8) | 139/299 (46.5) | 0.139 | |
| **Chronic comorbidities** |  |  |  | |  | |  |  |  | |
| Hypertension | 141/335(42.1) | 192/514(37.4) | 0.096 | |  | | 122/299 (40.8) | 113/299 (37.8) | 0.061 | |
| Diabetes | 87/335(26.0) | 149/514(29.0) | 0.069 | |  | | 80/299 (26.8) | 80/299 (26.8) | <0.001 | |
| CHF | 56/335(16.7) | 95/514(18.5) | 0.047 | |  | | 53/299 (17.7) | 50/299 (16.7) | 0.027 | |
| COPD | 70/335(20.9) | 107/514(20.8) | 0.002 | |  | | 64/299 (21.4) | 65/299 (21.7) | 0.008 | |
| Malignant neoplasm | 34/335(10.1) | 68/514(13.2) | 0.102 | |  | | 33/299 (11.0) | 31/299 (10.4) | 0.022 | |
| Leukemia/lymphoma | 14/335(4.2) | 24/514(4.7) | 0.024 | |  | | 14/299 (4.7) | 11/299 (3.7) | 0.050 | |
| Hepatobiliary disease | 156/335(46.6) | 312/514(60.7) | 0.283^***^ | |  | | 147/299 (49.2) | 153/299 (51.2) | 0.040 | |
| Chronic renal disease | 54/335(16.1) | 110/514(21.4) | 0.143 | |  | | 52/299 (17.4) | 58/299 (19.4) | 0.053 | |
| **Acute comorbidities** |  |  |  | |  | |  |  |  | |
| Stroke | 27/335(8.1) | 29/514(5.6) | 0.089 | |  | | 22/299 (7.4) | 23/299 (7.7) | 0.012 | |
| Sepsis/septicemia | 145/335(43.3) | 306/514(59.5) | 0.327^***^ | |  | | 134/299 (44.8) | 145/299 (48.5) | 0.074 | |
| Acute pancreatitis | 66/335(19.7) | 66/514(12.8) | 0.172^**^ | |  | | 49/299 (16.4) | 43/299 (14.4) | 0.050 | |
| Acid-based unbalance | 167/335(49.9) | 310/514(60.3) | 0.209^**^ | |  | | 153/299 (51.2) | 164/299 (54.8) | 0.073 | |
| CHD | 51/335(15.2) | 82/514(16.0) | 0.020 | |  | | 46/299 (15.4) | 49/299 (16.4) | 0.028 | |
| Ascites | 72/335(21.5) | 164/514(31.9) | 0.253^**^ | |  | | 67/299 (22.4) | 72/299 (24.1) | 0.041 | |
| **Vital signs** |  |  |  | |  | |  |  |  | |
| CVP (mmHg) | 12(8-14) | 13(9-16) | 0.168^*^ | |  | | 12(8-14) | 12(9-16) | 0.072 | |
| Heart rate (bpm) | 98(85-112) | 96(82-109) | 0.080 | |  | | 98(85-112) | 96(81-107) | 0.101 | |
| Temperature (°C) | 36.9(36.5-37.4) | 36.8(36.5-37.3) | 0.070 | |  | | 36.9(36.5-37.4) | 36.8(36.5-37.2) | 0.080 | |
| Respiratory rate (bpm) | 21(18-24) | 21(18-24) | 0.058 | |  | | 21(17-24) | 21(18-24) | 0.029 | |
| Urine output (ml) | 1469 (602-2014) | 991(285-1347) | 0.402^***^ | |  | | 1372(553-1760) | 1187(371-1624) | 0.156 | |
| **Laboratory tests** |  |  |  | |  | |  |  |  | |
| WBC | 15.9(9.7-20.0) | 17.5(10.3-21.8) | 0.176^*^ | |  | | 15.7(9.6-19.8) | 16.6(10.2-20.1) | 0.103 | |
| Platelet count | 219(124-282) | 204(111-261) | 0.126 | |  | | 212(120-279) | 212 (121-268) | <0.001 | |
| Hemoglobin | 11.8(10.2-13,1) | 11.4(9.6-12.9) | 0.187^*^ | |  | | 11.7(10.1-13.0) | 11.6(9.7-13.1) | 0.049 | |
| Creatinine | 2.1(1.0-2.4) | 2.5(1.3-3.1) | 0.071 | |  | | 2.3(1.0-2.5) | 2.2(1.2-2.7) | 0.019 | |
| BUN | 33.0(17-40) | 42.3(22-53) | 0.359^***^ | |  | | 34.3(17-42) | 38.9(20-48) | 0.175 | |
| Albumin | 3.0(2.6-3.4) | 2.9(2.5-3.3) | 0.116 | |  | | 3.0(2.6-3.3) | 3.0(2.5-3.4) | 0.014 | |
| ALT | 61(24-478) | 50(23-247) | 0.107 | |  | | 60(24-473) | 49(29-372) | 0.006 | |
| AST | 134(44-686) | 111(44-499) | 0.055 | |  | | 135(44-622) | 118(42-538) | 0.007 | |
| Total bilirubin | 1.2(0.6-3.3) | 1.8(0.7-5.4) | 0.280^***^ | |  | | 1.2(0.6-3.7) | 1.4(0.6-3.7) | 0.077 | |
| INR | 1.5(1.2-2.0) | 1.8(1.4-2.5) | 0.187^**^ | |  | | 1.5(1.3-2.0) | 1.8(1.4-2.3) | 0.079 | |
| PT | 16.4(13.8-21.3) | 20.2(15.7-26.9) | 0.139^*^ | |  | | 16.6(13.9-21.6) | 19.2(15.2-25.2) | 0.077 | |
| Fibrinogen | 345(302-470) | 339(195-453) | 0.034 | |  | | 345(194-479) | 345(201-464) | 0.003 | |
| CK-MB | 1(1-4) | 1(1-5) | 0.024 | |  | | 1(1-4) | 1(1-5) | 0.016 | |
| Glucose | 204(130-226) | 189(126-220) | 0.099 | |  | | 203(133-225) | 194(130-221) | 0.065 | |
| Calcium | 7.5(7.0-8.2) | 7.4(6.8-8.0) | 0.116 | |  | | 7.5(6.9-8.2) | 7.5(6.9-8.1) | 0.016 | |
| Potassium | 4.2(3.6-4.6) | 4.3(3.7-4.7) | 0.137 | |  | | 4.2(3.6-4.6) | 4.2(3.6-4.7) | 0.048 | |
| Sodium | 137(134-140) | 136(133-139) | 0.132^*^ | |  | | 137(133-140) | 137(133-140) | 0.001 | |
| PCO2 | 50(42-56) | 50(40-57) | 0.009 | |  | | 50(41-56) | 50(41-57) | 0.029 | |
| PaO2/FiO2 ratio | 154(86-193) | 144(83-187) | 0.114 | |  | | 154(86-193) | 148(86-196) | 0.068 | |

**Table S5** Comparisons of baseline characteristics between the APP original cohort and PSM cohort. **Abbreviation:** SMD standardized mean difference. * means p value＜0.05, ** means p value＜0.01, *** means p value ＜0.001

| **Model name** | **AUC (CI)** | | | **ACC (CI)** | | |
| --- | --- | --- | --- | --- | --- | --- |
|  | **Confirmed** | **Suspected** | **Mean** | **Confirmed** | **Suspected** | **Mean** |
| Cat Boost Classifier | 0.80  [0.74, 0.84] | **0.77**  **[0.72, 0.81]** | **0.79** | **0.78**  **[0.73, 0.83]** | **0.72**  **[0.68, 0.76]** | **0.77** |
| Light Gradient Boosting | 0.75  [0.70, 0.79] | 0.75  [0.70, 0.80] | 0.75 | 0.77  [0.72, 0.82] | 0.75  [0.71, 0.78] | 0.76 |
| Support Vector Machine | **0.81**  **[0.75, 0.86]** | 0.73  [0.68, 0.77] | 0.77 | 0.76  [0.71, 0.81] | 0.73  [0.69, 0.77] | 0.75 |
| Random Forest Classifier | 0.77  [0.72, 0.82] | 0.76  [0.71, 0.81] | 0.77 | 0.77  [0.72, 0.82] | 0.73  [0.69, 0.77] | 0.75 |
| Logistic Regression | **0.81**  **[0.76, 0.86]** | 0.76  [0.71, 0.80] | **0.79** | 0.71  [0.65, 0.77] | 0.70  [0.66, 0.74] | 0.71 |

**Table S6** Performance of different models in internal validation. best performance was identified with bold.
